# Mitochondria-targeted heme oxygenase-1 induces oxidative stress and mitochondrial dysfunction in macrophages, kidney fibroblasts and in chronic alcohol hepatotoxicity^☆^

**DOI:** 10.1016/j.redox.2013.07.004

**Published:** 2013-07-23

**Authors:** Seema Bansal, Gopa Biswas, Narayan G. Avadhani

**Affiliations:** The Department of Animal Biology and the Mari Lowe Center for Comparative Oncology, School of Veterinary Medicine, University of Pennsylvania, Philadelphia, PA 19104, USA

**Keywords:** HO-1, Heme Oxygenase-1, ROS, Reactive Oxygen Species, NPR, NADPH cytochrome P 450 reductase, CcO, cytochrome c oxidase, ER, Endoplasmic reticulum, DCFH-DA, Dichlorofluorescein diacetate, Heme oxygenase-1, Mitochondrial targeting, Cytochrome c Oxidase, Heme aa3 content, ROS production, Autophagy

## Abstract

The inducible form of Heme Oxygenase-1 (HO-1), a major endoplasmic reticulum (ER) associated heme protein, is known to play important roles in protection against oxidative and chemical stress by degrading free heme released from degradation of heme proteins. In this study we show that induced expression of HO-1 by subjecting macrophage RAW-264.7 cells to chemical or physiological hypoxia resulted in significant translocation of HO-1 protein to mitochondria. Transient transfection of COS-7 cells with cloned cDNA also resulted in mitochondrial translocation of HO-1. Deletion of N-terminal ER targeting domain increased mitochondrial translocation under the transient transfection conditions. Mitochondrial localization of both intact HO-1 and N-terminal truncated HO-1 caused loss of heme aa-3 and cytochrome c oxidase (CcO) activity in COS-7 cells. The truncated protein, which localizes to mitochondria at higher levels, induced substantially steeper loss of CcO activity and reduced heme aa3 content. Furthermore, cells expressing mitochondria targeted HO-1 also induced higher ROS production. Consistent with dysfunctional state of mitochondria induced by HO-1, the mitochondrial recruitment of autophagy markers LC-3 and Drp-1 was also increased in these cells. Chronic ethanol feeding in rats also caused an increase in mitochondrial HO-1 and decrease in CcO activity. These results show that as opposed to the protective effect of the ER associated HO-1, mitochondria targeted HO-1 under normoxic conditions induces mitochondrial dysfunction.

## Highlights

• Under hypoxia, the inducible Heme Oxygenase-1 (HO-1) is localized in mitochondria.
• N-terminal truncated HO-1 is more efficiently translocated to mitochondria.
• Mitochondria targeted HO-1 induces oxidative stress and CcO dysfunction.
• Mitochondrial HO-1 content is increased in alcohol treated rat livers.

## Graphical abstract

**Figure:**
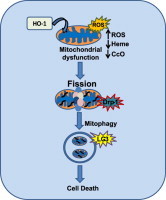

## Introduction

Heme oxygenases (HO) represent a family of evolutionarily conserved endoplasmic reticulum (ER) enzymes that have been described as fonts of multiple messengers [1]. HO's are widely considered as the central components of mammalian stress response and defense against oxidative stress [2], [3], [4], [5]. Three different isoforms of HO have been described in mammalian systems including the inducible HO-1; constitutive HO-2; and a newly identified HO-3, which is not catalytically active [6], [7]. Although its function remains obscure, HO-3 may be involved in heme binding or heme sensing [8]. Out of the three isoforms, the inducible HO-1 is highly concentrated in tissues that are heavily involved in the catabolism of heme proteins [9]. The HO's catalyze the oxidative cleavage of protoheme to biliverdin, liberating CO and free iron. The enzyme requires NADPH–cytochrome–P450-reductase (NPR) as the donor of electrons for substrate metabolism by HO-1[10], [11], [12].

The human HO-1 is comprised of a protein fold that primarily contains α-helices. The heme is held between two of these helices. The HO-1 acts as the cytoprotective stress protein, and provides defense against oxidative stress by accelerating the degradation of pro-oxidant heme and hemoproteins to the radical scavenging bile pigments, biliverdin and bilirubin [13], [14], [15], [16]. This protein is also induced in response to variety of stimuli such as free iron, inflammation, heavy metals, UV radiation and various oxidative stress conditions including hypoxia or conditions that produce ROS [4], [5], [17], [18], [19], [20], [21]. Under oxidative injury in some tissues heme-derived Fe and CO may exacerbate intracellular oxidative stress and cellular injury by promoting free radical generation in mitochondria and other cellular compartments [22], [23]. HO-1 overexpression is also known to promote mitochondrial sequestration of non-transferrin iron and induce macroautophagy contributing to the pathological iron deposition and bioenergetic failure in age related neurodegenerative disorders [24], [25], [26], [27], [28], [29], [30], [31], [32].

Studies also suggest the contribution of oxidative stress, chemical stress and Reactive Oxygen Species (ROS) in inducing the expression of HO-1. A study by Han et al. [33] suggested that mitochondria-derived H_2_O_2_ plays an important role in the intracellular signaling pathways, leading to up-regulation of HO-1 transcription in cultured endothelial cells. Some studies also suggested that increased intramitochondrial heme and subsequent ROS generation may be the driving force for mobilizing HO-1 in mitochondria [34].

In this study we examined the fate of induced HO-1 in macrophages exposed to physiological or chemical hypoxia. We have found that HO-1 is not only significantly induced but also a substantial portion of the induced protein is localized inside mitochondria. We further analyzed the N-terminal sequence motifs of the protein and found that a higher percentage of expressed N-terminal 16 amino acid lacking (ΔN16) protein is localized to mitochondria. An important consequence of mitochondria targeted HO-1 is the formation of shortened mitochondrial fragments as seen by immunocytochemistry, indicative of cellular toxicity and mitochondrial fission. Increased mitochondrial localization of HO-1 also induced inhibition of cytochrome c oxidase (CcO) activity and caused higher production of ROS. The mitochondria-targeting of HO-1 also promotes autophagy as evident by increased mitochondrial localization of LC3 and Drp-1. These results show that HO-1 induces mitochondrial dysfunction, and cellular pathology under certain growth conditions.

## Materials and methods

### Source of antibodies

Polyclonal antibody against human HO-1 (anti-rabbit) was purchased from Life Span Biosciences Inc., Seattle, WA. Antibody to human CcO subunit 1 (anti-mouse) was from Abcam, Cambridge, MA. Antibodies against human NPR (anti-mouse) and human actin (anti-goat) were from Santa Cruz Biotech., Santa Cruz, CA. Antibody to human dynamin related protein, Drp-1 was from BD Biosciences, San Jose, CA, USA and Microtubule-associated protein 1A/1B-light chain 3, LC-3 was from MBL International, Woburn, MA. Mitotracker green was purchased from Life Technologies, Grand Island, NY

### Cell culture conditions, exposure to hypoxia and CoCl_2_ treatment

RAW 264.7 mouse monocyte macrophages were cultured in Dulbecco's modified Eagles medium (DMEM) supplemented with 10% heat inactivated fetal calf serum and 100 μg/ml penicillin–streptomycin. Cells were grown under normal oxygen condition of 148 Torr or 21% O_2_. Cells grown up to 90% confluence under normoxia were latter exposed to hypoxia for 12 and 24 h. Simulation of realistic in vivo hypoxia requires that O_2_ tension be maintained at less than 5 Torr. This hypoxic condition was achieved in a temperature controlled hypoxic chamber by a constant flow of premixed gas that was certified to contain 1 Torr of oxygen and 5% CO_2_ (BOC gases, Murray Hill, NJ). For chemical hypoxia, cells grown to 70% confluence were treated with 150 μM CoCl_2_ in fresh medium and incubated for 0–96 h.

### Construction of plasmids

Full length mouse HO-1 (WT) cDNA was amplified from RNA from CoCl2 treated RAW 264.7 cells by reverse transcription followed by overlap PCR. N-terminal 16 and 33 amino acid coding region cDNA constructs (ΔN16 and ΔN33, respectively) were generated by PCR amplification of the parent cDNA using appropriate sense primers containing an ATG codon and upstream Kozak sequence. All constructs were engineered to contain 5′ Hind III and a 3′ Xba I sites and cloned in PCMV4 vector. The sequence properties of all the plasmid constructs were verified prior to use. The primers used for generating WT and mutant HO-1 are listed in Table 1.

**Table 1:** Primers used for generation of WT HO-1 and mutant constructs.

**Constructs**	**Primer**
**WT HO-1**	**FP:** ATCGGTACCACCGCCGTGATGGAGCGTCCACAGCCCGACAGCATG
	**RP:** ATCTCTAGATTACATGGCATAAATTCCCACTGCCACTGTTG
**ΔN16**	**FP:**ATCGGTACCACCGCCATGTTGAAGGAGGCCACCAAGGAGGTACACATC
**ΔN33**	**FP:** ATCGGTACCACCGCCATGAAGAACTTTCAGAAGGGTCAGGTGTCC

### Predictions of subcellular targeting

The Bioinformatics program, WoLF PSORT, which is an extension of the PSORT II program, converts protein amino acid sequences into numerical localization features and uses the *k* nearest neighbor classifier (kNN) to predict localization sites. This program was used to predict the putative mitochondrial targeting efficiency of the WT and N-terminal deletion HO-1 constructs.

### Transient transfection of WT and mutant HO-1 in COS-7 cells

COS-7 cells were grown in high glucose, Dulbecco's modified Eagle's medium (DMEM) supplemented with 10% heat inactivated fetal bovine serum (FBS) and 0.1% gentamicin. Cells were transiently transfected with WT, ΔN16 and ΔN33 cDNA's using FUGENE HD (Roche Diagnostics, Mannheim, Germany) transfection reagent. The transfection reagent/DNA ratio was maintained at 3:2 and after 48 h, the cells were harvested, washed in 1× phosphate buffered saline (137 mm NaCl, 2.7 mm KCl, 8.1 mm Na_2_HPO_4_, 1.5 mm KH_2_PO_4_, pH 7.4), and the cell pellets were used for further analyses.

### Isolation of subcellular fractions from COS-7 and RAW 264.7 cells

Cells were washed twice with ice cold phosphate buffered saline (PBS, 137 mm NaCl, 2.7 mm KCl, 8.1 mm Na_2_HPO_4_, 1.5 mm KH_2_PO_4_, pH 7.4 ) and lysed in RIPA buffer (25 mm Tris–HCl, ph 7.4, 150 mm NaCl, 0.1 mM EDTA, 1% Nonidet P-40, 0.1% deoxycholate, 0.025% NaN_3_, 1% protease inhibitor cocktail) to prepare cellular extract. Mitochondria and microsome fractions were isolated as previously described [35] with little modifications. Briefly, cells were resuspended in sucrose–mannitol buffer (20 mm Hepes, pH 7.5, containing 70 mm sucrose, 220 mm mannitol and 2 mm EDTA) and homogenized using a glass/Teflon Potter Elvehjem homogenizer (Wheaton Industries, Millville, NJ, USA) for approximately 30 strokes. The homogenate was centrifuged at 600×*g* for 10 min followed by another spin at 650×*g* for 10 min to remove nuclei and cell debris. The post-nuclear supernatant was then centrifuged at 8000×*g* for 15 min to sediment the crude mitochondrial fraction. The pellet was resuspended in sucrose–mannitol buffer, layered over a 1.0 m sucrose cushion and centrifuged at 8500×g for 20 min to purify the mitochondria. The purified mitochondria were washed with sucrose–mannitol buffer twice. The post-mitochondrial supernatant was centrifuged at 100,000×*g* to pellet microsomes. Mitochondria and microsomes were re-suspended in 50 mm potassium phosphate buffer (pH 7.5) containing 20% glycerol (v/v), 0.1 mm EDTA, 0.1 mm dithiothreitol (DTT) and 0.1 mm phenylmethylsulfonyl fluoride (PMSF). Total protein concentrations were determined using Lowry's method [36].

### SDS-PAGE and western blotting

Equal protein masses (50 μg) of crude cell lysates, mitochondria and microsomes were solubilized in Laemmli sample buffer, resolved on SDS-PAGE and transferred to nitrocellulose membranes. Membranes were probed with the indicated primary antibodies, followed by the appropriate HRP-conjugated secondary antibodies or IR-conjugated antibodies. Immunoreactive bands were developed with either chemiluminescence kit (Pierce) and developed in Biorad Analyzer or when probed with IR-conjugated antibodies visualized in Odyssey Licor, LICOR Biosciences, Lincoln, NE, USA.

### Spectrometric analysis of cytochrome c oxidase activity and heme aa3 content

CcO activity was measured by incubating 10 μg of freeze-thawed mitochondria prepared from transfected cells expressing WT and mutant HO-1 constructs in 1 ml of assay medium (25 mM potassium phosphate, pH 7.4, containing 0.45 mM dodecyl maltoside and 15 μM reduced cytochrome *c*) and measuring the decrease in absorbance at 550 nm due to cytochrome *c* oxidation. First order rate constants were measured and the amount of cytochrome c oxidized was calculated using an extinction coefficient of 21.1 mM^−1^cm^−1^ at 550 nm [37].

For measuring heme content, isolated mitochondria from mock, WT, ΔN16 cells equivalent to 900 μg of protein were incubated on ice for 30 min in 2 ml of 25 mM phosphate buffer, pH 7.4, containing 2% dodecyl maltoside before being split into two cuvettes. Sodium ascorbate (10–20 mg) was added to one of the cuvettes and after 10 min of incubation, the reduced minus oxidized difference spectra from 400 to 700 nm were recorded at room temperature (25 °C). The heme aa3 content was calculated from the difference spectra (ascorbate reduced minus air oxidized) using an absorption coefficient of 164 mM^−1^ cm^−1^ at 445 nm [38].

### ROS measurement

The ROS measurement was based on the principle that upon entry into cells, DCFH-DA (Molecular Probes, Eugene, OR, USA) is cleaved by intracellular esterases to form non-fluorescent 2′,7′-dichlorfluorescein, DCFH, which is then oxidized by peroxides to highly fluorescent DCF. COS-7 cells were transfected with intact WT and N-terminal deletion variants. As controls, cells were also treated with membrane permeable SOD, catalase and N-acetyl cysteine, NAC (25 mM). 48 h post transfection, the media was aspirated and the cells were rinsed with 1X PBS. The cells were loaded with 15 μM DCFH-DA for 15 min in the dark to allow intracellular conversion of DCFH. At the end of incubation, cells were scraped off gently in 1 ml ice cold PBS. 2×10^6^ cells in 1 ml of PBS were incubated and fluorescence was recorded using LPS-220B spectroflourometer (Photon Technology International, Bermingham, NJ) at an excitation wavelength of 485 nm and emission wavelength of 535 nm (for 20 min). The differences between the end points and the start points were used to calculate the DCF fluorescence units.

### Immunofluorescence microscopy

Immunofluorescence microscopy was carried out with 0.1% Triton X-100 permeabilized cells as described before [39] using primary HO-1 (anti-rabbit), CcO1 (anti-mouse), LC-3 (anti-mouse) and Drp1 (anti-mouse) antibody at 1:100 dilutions each. The cells were then stained with 1:300 dilution of Alexa 488-conjugated anti-rabbit antibody and Alexa 594-conjugated anti-mouse IgG (Molecular Probes, Inc., Eugene, OR). Cells were also stained with 300 nM Mitotracker Green (Molecular Probes, Inc., Eugene, OR) for 30 min at 37 °C to stain mitochondria. Slides were viewed through a Leica TCS SP5 Confocal Microscope, and Pearson′s coefficient for co-localization was calculated using Volocity software 5.3.

### Animal feeding experiments

Sprague-Dawley rats (about 150 g) were fed with alcohol for 2, 4, 6, 8, and 10 weeks, and pair-fed controls received isocaloric diet. The standard procedure for alcohol feeding was based on the Lieber De Carli protocol [40]. Animals were fed ad libitum a nutritionally balanced liquid diet containing 2–36% caloric equivalent of ethanol, 18% protein, 35% fat, and 11% carbohydrate as % of total calories (supplied by BioServe Corp., San Diego). The ethanol content was steadily increased from a caloric equivalent of 2–4% during the 1st week to 36% by the 4th week and then maintained at this level until 10 weeks. Control pair-fed animals received the same diet except that alcohol was isocalorically replaced by maltose dextrins. Feeding was carried out in the Animal Resource Facility of Thomas Jefferson University Medical College, Philadelphia, under their approved animal care protocol.

### Statistical analysis

Data are presented as mean±SEM. The Student's *t* test was used for comparisons between the groups. Statistical significance of value *p*<0.05 was considered significant.

## Results

### Mitochondrial localization of hypoxia induced HO-1 in cultured cells

**Fig. 1:**
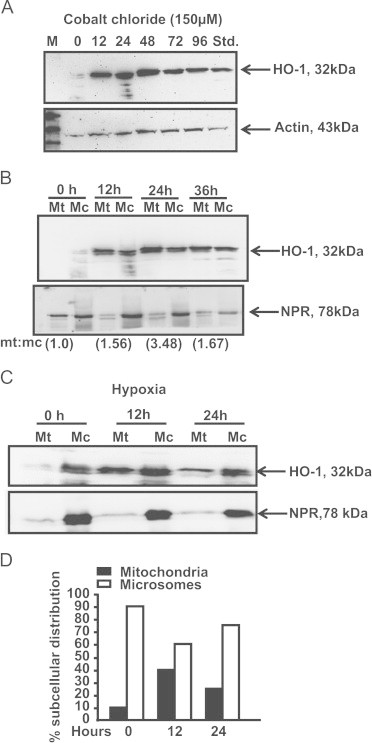
Hypoxia and CoCl_2_ induced HO-1 localizes to mitochondria. (A) RAW 264.7 cells were treated with CoCl_2_ for 0–96 h. Whole cell lysates (50 μg each) were prepared and subjected to immunoblot analysis using HO-1 antibody. Actin served as loading control. (B). Mitochondria and microsomes were prepared from cells treated with CoCl_2_ for 0, 12, 24 and 36 h. The proteins (50 μg each) were resolved on SDS-PAGE and the immunoblot was developed with antibody to HO-1 (1:1500 dilution). The blot was also co-developed with antibody to NPR (1:2500 dilution) to detect cross-contamination. (C) Mitochondrial and microsome proteins from RAW 264.7 cells exposed to hypoxia (1% O_2_) for 0, 12 and 24 h were resolved on SDS-PAGE and probed for HO-1 expression. 50 μg protein was used in each case. The purity of mitochondrial isolates was assessed by reprobing the blot with microsomal specific marker, NPR. (D) Histogram represents the % subcellular distribution of HO-1 protein in the mitochondria and microsomes after hypoxia treatment.

The RAW 264.7 macrophages were exposed either to hypoxia (1% O_2_) for 12 and 24 h or treated with 150 μM CoCl_2_ for 12, 24, 48, 72 and 96 h as indicated. The immunoblots of cell lysates showed a time dependent increase in total cellular HO-1 protein up to 48 h followed by a steady decline up to 96 h (Fig. 1A). Similarly, the mitochondrial and microsomal distribution of protein showed a time dependent increase of mitochondrial HO-1 with a maximum at 24 h which was accompanied by a gradual decrease in microsomal HO-1 (Fig. 1B). The values in parentheses at the bottom of the immunoblot show ratios of mitochondrial:microsomal HO-1 protein. Exposure of cells to hypoxia also led to a 2–4 fold induction of HO-1 in mitochondria as well as in the microsomes; the mitochondrial HO-1 peaked at 12 h while the microsomal HO-1 levels remained high until 24 h of hypoxia (Fig. 1C and D). The level of microsomal contamination in the mitochondrial preparation was minimal as judged by the levels of NPR protein in mitochondrial preparations (Fig. 1B and C). The blot for total cell lysate was probed with actin as a loading control (Fig. 1A).

**Fig. 2:**
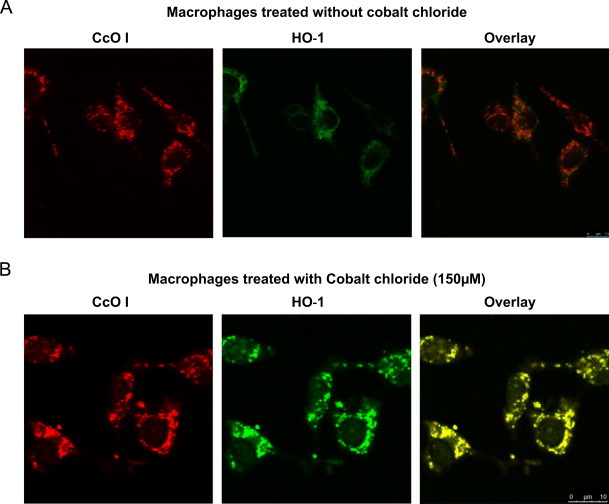
Immunocytochemical localization of HO-1 in mitochondria: (A) and (B) RAW 264.7 cells without treatment (A) and with 150 μM CoCl_2_ (B) for 48 h were stained with antibody to mitochondria specific marker, Cco I and antibody to HO-1. The cells were subsequently incubated with Alexa 488-conjugated anti-rabbit antibody and Alexa 594-conjugated anti-mouse goat IgG for colocalization of fluorescence signals. Slides were examined by confocal microscopy through Leica TCS SP5 microscope.

The localization of HO-1 in mitochondria was further investigated by immunocytochemical analysis of cells treated with 150 μM CoCl_2_. Cells were subjected to double immunostaining with HO-1 antibody and antibody to mitochondrial specific marker CcO I (Fig. 2A and B). When compared with the normal mitochondrial pattern in the untreated cells, approximately 90% of cobalt chloride treated cells showed a robust colocalization with CcO I stained organelles (Fig. 2B). Notably, in CoCl_2_ treated cells, the mitochondrial pattern exhibited a granulated punctate structures compared to elongated mitochondria structures in control cells (Fig. 2A).

**Fig. 3:**
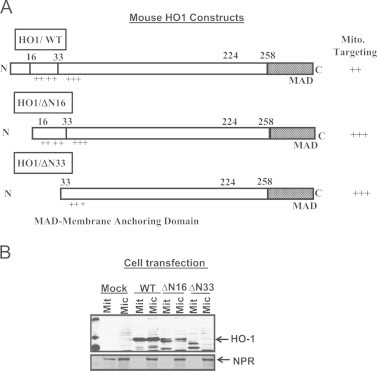
Mitochondrial targeting of HO-1 protein: (A) Cartoon depicts the targeting domains of WT and truncated (ΔN16 and ΔN33) HO-1 cDNA's. The cDNA were cloned in PCMV4 using Hind 3 and Xba I restriction sites at 5′ and 3′ termini, respectively. The N-terminal 16 and 33 amino acids were deleted in ΔN16 and ΔN33, respectively. The ++ and +++ annotations on the extreme right represent the arbitrary units of mitochondrial targeting efficiencies. Mitochondrial and microsomal proteins from cells transfected with Mock, WT and N-terminal deletion mutant constructs cDNA were resolved on SDS-PAGE and probed for HO-1 expression. The purity of the mitochondrial isolates was assessed by reprobing the blot with microsomal specific marker, NPR.

Since HO-1 was induced by hypoxia and was found to be targeted to mitochondria, we analyzed the amino acid sequence and observed that it consists of clusters of positive charges at the N-termini (Fig. 3A). We therefore generated progressive N-terminal deletion constructs as shown in Fig. 3A to assess the sequence regions important for mitochondrial targeting. The WoLF PSORT program was used to determine the putative targeting efficiencies of these proteins. As shown in Table 2, the computer based prediction for mitochondrial targeting potential is higher when the N-terminal hydrophobic (1–16 amino acids) and hydrophilic (16–33 amino acids) amino acid stretches were deleted. The ++ and +++ notations in Fig. 3A represent arbitrary units of targeting efficiencies.

**Table 2:** Prediction of distribution of WT HO-1 and mutants into various subcellular organelles using WOLFPSORT.

Constructs	Subcellular organelles			
	Mitochondria	Cytosol	Nucleus	ER

WT	3.0	13.0	2.0	10.0
ΔN16	12.5	13.5	8.5	4.3
ΔN33	12.0	3.0	–	8.7

The wild type and deletion constructs cloned in mammalian expression vector PCMV4 were transiently transfected into COS-7 cells (Fig. 3B). Forty eight hours post-transfection, the subcellular fractions were prepared and the level of HO-1 was determined by immunoblot analysis (Fig. 3B). The mock transfected cells did not show any significant amount of protein in either mitochondria or microsomes. In the transfected cells, nearly 50% of ectopically expressed WT HO-1 (HO-1/WT) protein was localized to the mitochondrial fraction and the remaining 50% in the microsomal fraction. The N-terminal 16 amino acid truncated (HO1/ΔN16) protein showed a significantly higher mitochondrial localization and a lower level of ER targeting. The N-terminal 33 amino acid deletion construct (HO1/ΔN33) showed negligible ER targeting but a prominent mitochondria targeting. The faster migrating bands in all three cases probably represent non-specific proteolytic products. These results show that ectopically expressed HO-1 is targeted to mitochondria and the N-terminal truncation markedly reduced ER targeting but increased mitochondria targeting.

### Cytochrome c oxidase activity and heme aa3 contents are diminished by increased mitochondrial targeting of HO-1

**Fig. 4:**
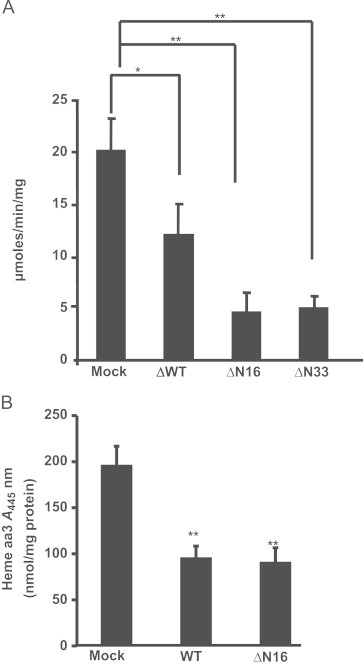
Measurement of Cytochrome c oxidase activity and heme aa3 contents: (A) CcO activity was measured by incubating 10 μg of freeze-thawed mitochondrial extract from cells transfected with Mock, WT, ΔN16 and ΔN33 cDNA in 1 ml of assay medium (25 mM potassium phosphate, pH 7.4, containing 0.45 mM dodecyl maltoside and 15 μM reduced cytochrome c. The CcO activity was measured as described in the “Materials and methods”. (B) Mitochondrial proteins from mock, WT and ΔN16 transfected cells were solubilized in lauryl maltoside containing buffer and used for spectral analysis as described in the Materials and methods section. Difference spectra of reduced minus air oxidized samples were recorded in the range of 400–700 nm and heme aa3 contents were calculated also as described in the Materials and methods section. ^⁎⁎^ represents statistical significance of *p*<0.05.

We investigated the possible effects of mitochondria targeted HO-1 on mitochondrial function by assaying cytochrome c oxidase (CcO) activity and heme aa3 contents of mitochondria from transiently transfected cells. As seen in Fig. 4A, CcO activity was inhibited by ~40% in the mitochondria from cells expressing WT HO-1 protein, whereas about 75% inhibition was observed in cells expressing HO1/ΔN16 and HO1/ΔN33 proteins. The heme aa3 levels measured by the air oxidized vs ascorbate reduced difference spectra at 445 nm were significantly lower in cells transfected with WT HO-1 and HO1/ΔN16 (Fig. 4B). These results suggest that mitochondria targeted HO-1 induces heme degradation and also diminishes the activity of heme containing terminal oxidase, CcO.

### Increased ROS production by mitochondria targeted HO-1

**Fig. 5:**
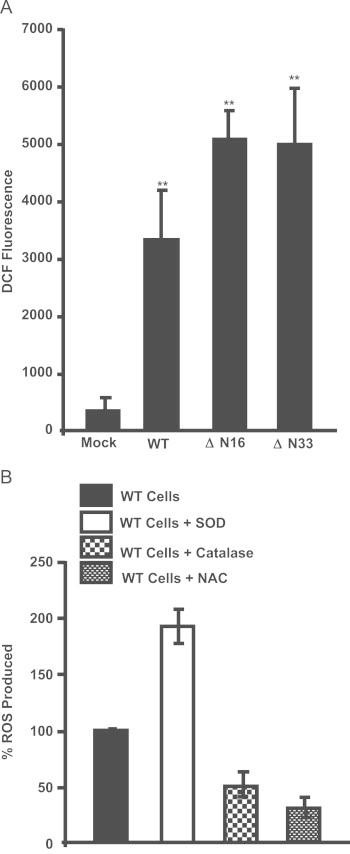
ROS production by mitochondria targeted HO-1 (A) ROS levels in mock, WT, ΔN16 and ΔN33 transfected cells were measured using DCFH-DA substrate. 48 h post transfection, the media was aspirated and the cells were rinsed with 1X PBS. The cells were loaded with 15 μM DCFH DA for 15 min in dark to allow intracellular conversion of DCFH. At the end of incubation, cells were scraped off gently in 1 ml ice cold PBS. 2×10^6^ cells in 1 ml of PBS were incubated and fluorescence was recorded using LPS-220B spectroflourometer (Photon Technology International, Bermingham, NJ) with an excitation wavelength of 485 nm and emission wavelength of 535 nm (for 20 min). As controls, cells were also treated with membrane permeable SOD (300 U/ml), catalase (200 U/ml) and N-acetyl cysteine, NAC (25 mM). ^⁎⁎^ indicates *p*<0.05.

Previously we and others showed that disruption of CcO complex by hypoxia, ischemia/reperfusion and alcohol toxicity adversely affected CcO activity [41], [42], [43], [44], [45], [46] and induced ROS production possibly because of disruption of respirosome supercomplexes [42], [43], [46]. In this study therefore, we evaluated the effects of mitochondria targeted HO-1 on mitochondrial ROS production. As seen in Fig. 5A, there was a nearly 8 fold increase in ROS production in cells transfected with WT HO-1 cDNA construct as measured by the DCFH-DA method. The level of ROS production was substantially higher in cells expressing HO1/ΔN16 and HO1//ΔN33 proteins, which cause more severe effect on CcO activity. DCFH-DA and other fluorescent probes used for free radical detection generally yield non-specific signals [47]. The specificity of the signal in our assays was ascertained using various controls shown in Fig. 5B. Treatment with cell permeable catalase and antioxidant N-acetyl cysteine markedly reduced the signal, while treatment with cell permeable SOD increased the signal in control cells suggesting that these cells produce substantial amount of O_2_^−^ which is converted to H_2_O_2_ by SOD treatment. These results together suggest that as opposed to the known cytoprotective effects of ER associated HO-1, the mitochondria targeted HO-1 induces oxidative stress.

### Immunocytochemical localization of HO-1 in mitochondria and induction of mitochondrial autophagy

**Fig. 6:**
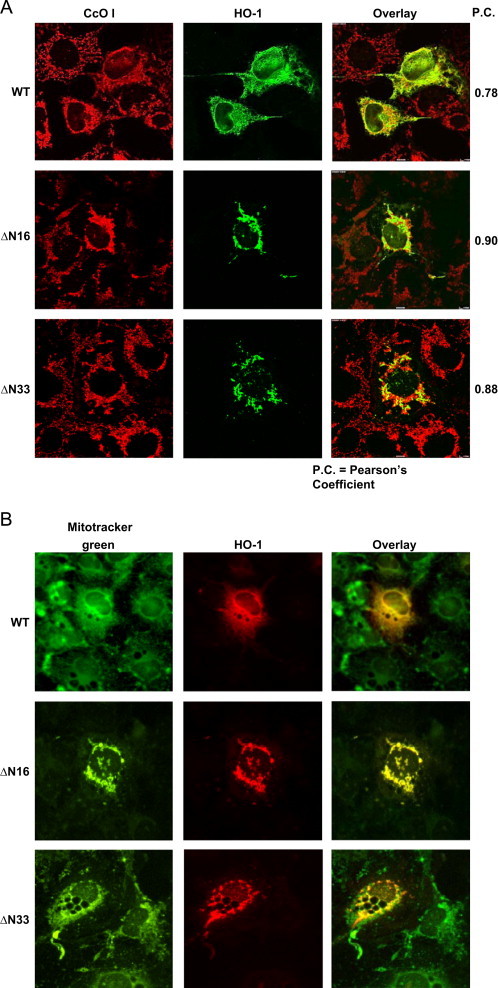
Intramitochondrial localization of HO-1: (A) Immunofluorescence microscopy was carried out with permeabilized Cos-7 cells transfected with WT, ΔN16 and ΔN33 cDNA's as described in the Materials and methods section. The cells were washed, blocked with 5% goat serum and incubated with primary HO-1 (anti-rabbit) antibody and mitochondria specific marker, CcO I (anti-mouse). The cells were subsequently incubated with Alexa 488-conjugated anti-rabbit antibody and Alexa 594-conjugated anti-mouse goat IgG for colocalization of fluorescence signals. (B) The transfected cells were also co-stained with mitotracker green for 30 min at 37 °C prior to imaging.

Mitochondrial localization of HO-1 in transiently transfected cells was further ascertained by immunochemical co-localization with mitochondria specific CcO I protein and mitotracker green (Fig. 6). As seen from Fig. 6A, cells transfected with WT HO-1 protein showed significant co-localization with mitochondrial CcO I antibody (Pearson's coefficient of 0.78). More intense colocalization was observed with N-terminal truncation (ΔN16 with a Pearson′s coefficient of 0.90 and ΔN33 with a Pearson's coefficient of 0.88). These results are consistent with the immunoblot analysis of proteins from transfected cells in Fig. 3. To further confirm the mitochondrial localization of HO-1 and to ascertain the identity of organelles being stained, we stained cells transfected with HO-1 constructs with Mitotracker green and HO-1 antibody. The staining pattern showed complete overlap of these HO-1 antibody stained, shortened mitochondrial filamentous structures with Mitotracker green (Fig. 6B). The co-localization of HO-1 with Mitotracker was more robust in cells transfected with ΔN16 and ΔN33 HO-1 constructs.

**Fig. 7:**
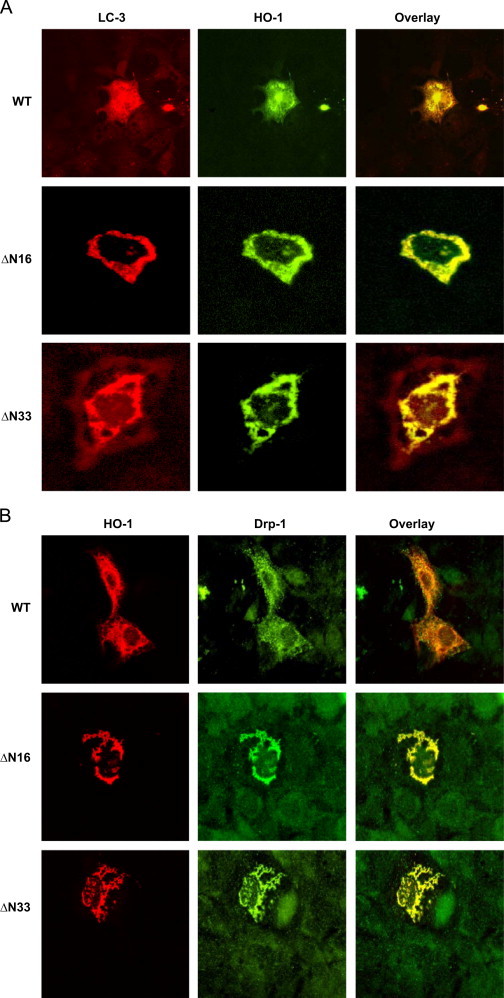
Induction of mitochondrial fission and autophagy: (A) and (*B*) The immunofluorescence microscopy was carried out with permeabilized Cos-7 cells transfected with WT, ΔN16 and ΔN33 cDNA's. Cells were incubated with primary HO-1 (anti-rabbit) antibody, and were co-stained with mitochondrial fission marker DRP-1 (A) and autophagy marker LC-3 (B) antibodies. The slides were subsequently stained with Alexa conjugated antibodies and examined through Olympus microscope.

Mitochondrial fission is a normal physiological process although excessive fission can be an indicator of abnormal mitochondrial dynamics [48], [49]. The effect of mitochondrial HO-1 expression on mitochondrial dynamics was investigated by immunofluorescence microscopy of cells stained with antibody to Drp-1, which is an indicator of fission and LC-3, which is an indicator of autophagy. Cells transfected with the three HO-1 constructs were stained with antibodies to mitochondria-specific protein, CcO 1 and HO-1. Since mitochondria targeted HO-1 induced granulated mitochondria instead of elongated punctate structures, we investigated the staining patterns with antibodies to Drp-1 and LC-3 proteins. Interestingly, cells expressing the N-terminal truncated proteins showed significant increase in the intensity of LC-3 punctate structures (Fig. 7A) and Drp-1 staining (Fig. 7B), which are in close association with fragmented/abnormal mitochondria. These results suggest that mitochondria-targeted HO-1 induces mitochondrial oxidative stress and mitochondrial autophagy.

### Mitochondrial HO-1 level in livers of rats fed with ethanol

**Fig. 8:**
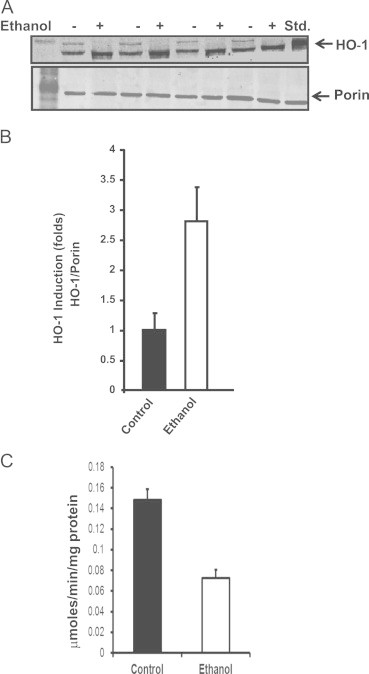
Mitochondrial HO-1 level in livers of rats fed with ethanol for 10 weeks: (A) Mitochondria were prepared from control rats and pair fed ethanol for 10 weeks using Lieber decarli diet. 50 μg mitochondrial protein each was subjected to immunoblot analysis using antibody to HO-1. The blot was also co-developed with mitochondrial specific marker, Porin as a loading control. (B) The HO-1 band intensities from controls and ethanol treated rats (*n*=4)were averaged using Image J and plotted. (C) CcO activity of rat liver mitochondria from control and pair-fed rats shown in (A) was measured as described in “Materials and methods”. Data are presented as±S.E. from three experiments, and groups were compared using an unpaired, two-tailed Student's *t* test. ^⁎⁎^ indicates *p*<0.05.

Several studies show that ethanol toxicity is associated with mitochondrial dysfunction and oxidative stress [39], [42], [46], [50], [51], [52], [53], [54]. Oxidative stress conditions also induce HO-1 expression. Although some studies suggest cytoprotective role of microsomal HO-1 in ethanol treated cells/tissues, it is unclear if HO-1 is also targeted to mitochondria under these conditions. The immunoblots of liver mitochondria from livers of rats subjected to chronic ethanol feeding for 10 weeks using the Lieber-De Carli liquid diet and pair fed controls (Fig. 8A) show a near 3 fold increase in mitochondrial HO-1 level as compared to control livers. Results also show a 40–50% lower CcO activity (Fig. 8C) suggesting that mitochondria-targeted HO-1 may also contribute to alcohol toxicity.

## Discussion

The heme oxygenases (HO) are mostly localized on the ER membrane through a single transmembrane anchoring domain present at the C-terminus of the protein [55]. The N-terminal end of the protein contains the ER targeting signal and the C-terminus contains the stop transfer domain which helps determine the membrane topology of the protein. Mitochondrial localization of HO-1 was first reported by Srivastava and Pandey in *Mastomys Coucha* during malaria infection [56], and subsequently reported by several other groups [32], [34]. However, the functional significance of mitochondria-localized HO-1 remains unclear. Here we report that induced expression of HO-1 in RAW-264.7 cells by treatment with CoCl_2_ or exposure to hypoxia resulted in the localization of HO-1 to mitochondria. Furthermore, transient transfection of COS-7 cells with full length and N-terminal truncated HO-1 cDNA constructs also resulted in the significant mitochondrial localization of HO-1. Consistent with what we observed with a number of inducible and constitutively expressed CYP proteins with chimeric N-terminal signals [57], deletion of N-terminal most ER targeting domain resulted in markedly increased mitochondrial targeting possibly by increasing the cystoplasmic pool of proteins available for mitochondrial translocation or by activating cryptic mitochondria targeting signal. Although we have not identified the precise mitochondria targeting signal in this study, the sequence stretch between 33 and 39 amino acids rich in positively charged residues possibly functions as a cryptic mitochondria targeting signal.

An important function of this monooxygenase is to breakdown free heme, a known oxidant, by oxidative cleavage of porphyrin ring to biliverdin with the release of Fe^3+^ ion and CO. Cellular stress causes an increase in hemoprotein turnover under oxidative or drug-induced hemolysis or drug/UV induced degradation of cytochrome P450, resulting in the accumulation of free heme [58], [59]. Thus, HO-1 plays a critical role in heme detoxification mechanism thereby preventing the accumulation of free heme in biological membranes and therefore help alleviating heme induced oxidative stress [5], [17]. Although majority of the published studies point to the putative anti-oxidant effects of HO-1 in different cell types and under different experimental conditions [13], [21], [34], [60] several studies suggest pro-oxidant properties of mitochondrial targeted HO-1 [5]. The HO reaction releases iron, which may lead to deleterious effects on iron reutilization and sequestration pathways. During the reaction HO potentially generates significant amount of H_2_O_2_ which can be a source of ^•^OH [61]. In vitro studies have also indicated that under certain conditions HO activity may potentiate, rather than abrogate oxidant toxicity [62], [63] by reversing the cytotoxic effects of H_2_O_2_ when the HO-1 activity is inhibited using specific inhibitors.

Increased HO mRNA expression and protein levels have been reported in a wide spectrum of diseases including neurodegenerative diseases such as Alzheimer's, Parkinson's, musculo-skeletal diseases, varieties of cancers, cardiac diseases and infection/inflammation [25], [27], [64], [65], [66]. Both cytotoxic and cytoprotective roles have been ascribed to HO overexpression in these diseases. Similar is the case with mitochondria-targeted HO-1. One study showed mitochondrial HO-1 induction in rat liver adversely affected the expression of mitochondria-targeted NOS and mitochondrial NO dependent oxidant yield [67]. Bindu et al. [34] reported that in gastric mucosal cells, mitochondrial oxidative stress induced accumulation of mitochondrial heme was alleviated by mitochondria targeted HO-1 suggesting a cytoprotective role. Slebos et al. [68] showed that in lung epithelial cells mitochondria targeted HO-1 rendered protection against cigarette smoke extract-induced mitochondrial membrane depolarization and loss of ATP. However, studies in transiently transfected primary rat neuroglial cells showed that mitochondria-targeted HO-1 induced oxidative mitochondrial damage [69]. Similarly in an endotoxin induced rat model of sepsis, mitochondrial HO-1 caused mitochondrial accumulation of free iron leading to mitochondrial dysfunction [70]. In a detailed study, Darley-Usmar's group showed that hemin caused mitochondrial respiratory and metabolic dysfunction and increased lipid peroxidation in bovine aortic endothelial cells [71]. In continuation of this study, recently this group showed targeted expression of chimeric HO-1 with fused N-terminal mitochondrial targeting signal rendered protection against hypoxia induced mitochondrial damage [60]. In the present study we show that ectopic expression of intact and N-terminal truncated HO-1 in Cos-7 cells caused loss of CcO activity, loss of heme aa3, increased ROS production and cell death. These contrasting effects of mitochondrial HO-1 probably reflect cell specific differences and the nature or extent of mitochondrial defense systems against oxidative stress. A common observation in most of the above studies is the loss of heme aa3 and loss of CcO activity.

We hypothesize that depending on the cell type, mitochondrial HO-1 induced changes in mitochondrial electron transport chain activity may drive them either towards apoptosis or mitophagy for inducing either cell death or biogenesis of new and healthy mitochondria. For example, during inflammation, the induction of HO-1 has been implicated as an inducer of autophagy leading to cell survival and anti-inflammatory effects and therefore the mechanism preserves the mitochondrial integrity through the activation of mitochondrial-selective autophagy (mitophagy) which enhances cell survival [72]. On the other hand, in models of neurodegeneration due to Parkinson's and Alzheimer's disease, overexpression of HO-1 leads to activation of apoptosis or autophagy without any significant biogenesis contributing to neuronal cell death. Our results on the overexpression HO-1 cDNA constructs by transient transfection in COS-7 cells also shows that induction of HO-1 in mitochondria contributes to CcO dysfunction and ROS production which is detrimental to mitochondrial function inducing autophagy. In a previous study we showed that hypoxia induced mitochondrial dysfunction in RAW264.7 cells [43]. In this study we show that hypoxia induced HO-1 expression and mitochondrial localization of HO-1 in RAW264.7 cells indicating a connecting link between Mito HO-1 content and mitochondrial dysfunction.

The possible link between mitochondrial HO-1 and loss of CcO activity was further supported by our results showing increased hepatic mitochondrial HO-1 in rats fed with chronic doses of alcohol using the Lieber-De Carli nutritionally balanced liquid diet [40]. These results are significant in view of our previous studies which marked loss of CcO activity and loss of supramolecular electron transport chain complexes in rats fed with ethanol for 10 weeks [42].

## Authors' contributions

SB and GB carried out the experiments and SB, GB, and NGA did the analysis and interpretation of data and wrote the manuscript.

## Submission declaration

This work has not been published previously or submitted elsewhere. This work was carried out in accordance with the Code of Ethics of the World Medical Association.
